# PCA3 in prostate cancer and tumor aggressiveness detection on 407 high-risk patients: a National Cancer Institute experience

**DOI:** 10.1186/s13046-015-0127-8

**Published:** 2015-02-06

**Authors:** Roberta Merola, Luigi Tomao, Anna Antenucci, Isabella Sperduti, Steno Sentinelli, Serena Masi, Chiara Mandoj, Giulia Orlandi, Rocco Papalia, Salvatore Guaglianone, Manuela Costantini, Giuseppe Cusumano, Giovanni Cigliana, Paolo Ascenzi, Michele Gallucci, Laura Conti

**Affiliations:** Clinical Pathology, Regina Elena National Cancer Institute, IRCCS, Via Elio Chianesi 53, 00144 Rome, Italy; Department of Sciences, University Roma Tre, Rome, Italy; Scientific Direction, Regina Elena National Cancer Institute, IRCCS, Rome, Italy; Department of Pathology, Regina Elena National Cancer Institute, IRCCS, Rome, Italy; Urology Department, Regina Elena National Cancer Institute, IRCCS, Rome, Italy; Interdepartmental Laboratory of Electron Microscopy, University Roma Tre, Rome, Italy

**Keywords:** Prostate cancer, Urine and blood biomarkers, Prostate Specific Antigen, Prostate Cancer gene 3, Tumor aggressiveness

## Abstract

**Background:**

Prostate cancer (PCa) is the most common male cancer in Europe and the US. The early diagnosis relies on prostate specific antigen (PSA) serum test, even if it showed clear limits. Among the new tests currently under study, one of the most promising is the prostate cancer gene 3 (*PCA3*), a non-coding mRNA whose level increases up to 100 times in PCa tissues when compared to normal tissues. With the present study we contribute to the validation of the clinical utility of the PCA3 test and to the evaluation of its prognostic potential.

**Methods:**

407 Italian men, with two or more PCa risk factors and at least a previous negative biopsy, entering the Urology Unit of Regina Elena National Cancer Institute, were tested for PCA3, total PSA (tPSA) and free PSA (fPSA and f/tPSA) tests. Out of the 407 men enrolled, 195 were positive for PCa and 114 of them received an accurate staging with evaluation of the Gleason score (Gs). Then, the PCA3 score was correlated to biopsy outcome, and the diagnostic and prognostic utility were evaluated.

**Results:**

Out of the 407 biopsies performed after the PCA3 test, 195 (48%) resulted positive for PCa; the PCA3 score was significantly higher in this population (*p* < 0.0001) differently to tPSA (*p* = 0.87). Moreover, the PCA3 test outperformed the f/tPSA (*p* = 0.01). The sensitivity (94.9) and specificity (60.1) of the PCA3 test showed a better balance for a threshold of 35 when compared to 20, even if the best result was achieved considering a cutoff of 51, with sensitivity and specificity of 82.1% and 79.3%, respectively. Finally, comparing values of the PCA3 test between two subgroups with increasing Gs (Gs ≤ 6 *versus* Gs ≥ 7) a significant association between PCA3 score and Gs was found (*p* = 0.02).

**Conclusions:**

The PCA3 test showed the best diagnostic performance when compared to tPSA and f/tPSA, facilitating the selection of high-risk patients that may benefit from the execution of a saturation prostatic biopsy. Moreover, the PCA3 test showed a prognostic value, as higher PCA3 score values are associated to a greater tumor aggressiveness.

## Background

Prostate cancer (PCa) is the most common malignancy in men of Western populations and one of the major burden in public health [1], despite numerous efforts were made attempting to clarify the various aspects of this disease [2-4]. During the last years an increasing PCa incidence has occurred, probably linked to the introduction of the prostate specific antigen (PSA) determination in terms of opportunistic screening [5]. The PSA test actually brought to the diagnosis of a high number of asymptomatic and preclinical forms of PCa, but it has not been associated with a decrease in mortality, opening a wide debate on the diagnostic utility of this test [6]. One of the main disadvantages of the PSA test is its low specificity, which causes the execution of a high percentage of negative biopsies (60-75%), especially in patients with total PSA (tPSA) levels between 4 and 10 ng/ml [7,8]. A great effort is therefore constantly turned to the research of new markers capable to improve the PCa diagnosis, to identify the asymptomatic and more aggressive forms and to reduce the number of biopsies, lowering the risk of pain, bleeding and infection to many patients [9]. Among the characterized biomarkers one of the most promising for its diagnostic potential, is the Prostate Cancer gene 3 (*PCA3*). *PCA3* (also known as *DD3* or *DD3PCA3*) is located on chromosome 9 and is transcribed into a non-coding prostate-specific mRNA which is overexpressed in tumor cells, from 60 to 100 times, when compared to the normal prostate tissue [10]. The PCA3 test is based on the quantification of the PCA3 mRNA on urine sample after digital-rectal examination (DRE), using the methodology of the transcription mediated amplification (TMA). The obtained result is then normalized to the amount of PSA mRNA, evaluated in the same urine sample, in order to calculate the PCA3 score (PCA3 mRNA/PSA mRNA × 1000). To date, many studies have been performed and most of them showed how the PCA3 test represents a useful tool to predict PCa, but questions about the optimal cutoff and the ability of PCA3 to predict tumor aggressiveness still remain highly controversial [11,12]. Here, we report the results of the PCA3 test among an Italian prospective cohort of high-risk PCa patients in order to evaluate its actual clinical utility as a diagnostic test additional and/or alternative to the PSA test. Moreover, best PCA3 cutoff was assessed to better discriminate patients with and without PCa. Finally, the correlation between the results of the PCA3 test and the tumor aggressiveness has been evaluated.

## Methods

### Patient selection

Between November 2009 and May 2011, 407 consecutive men with two or more risk factors for PCa and at least a previous negative biopsy entered the Urology Unit of Regina Elena National Cancer Institute. Risk factors for PCa could be: tPSA higher than 2,5 ng/ml, a family history of PCa, a borderline DRE and the presence of pre-neoplastic forms in a prior biopsy. None of the patients had a history for PCa and none was taking drugs able to lower PSA since at least one month. Biopsies evidencing pre-neoplastic forms, such as atypical acinar proliferation (ASAP), low-grade prostatic intraepithelial neoplasia (LGPIN) lesions or high grade PIN (HGPIN), were classified as negative. Once tests were carried out, patients were addressed more or less urgently towards a saturation prostatic biopsy. To date, all patients underwent a prostatic biopsy. This study was approved by the Ethics Committee of Regina Elena National Cancer Institute and a written informed consent was obtained from all participants.

### Sample processing

Blood samples were collected in tubes containing gel and clot activator for serum separation (Vacutainer, Becton-Dickinson, Franklin Lakes, NJ, USA). Samples were centrifuged within 1 h at 2500 g for 15 min and stored in aliquots at −80°C until processing. Serum tPSA and fPSA were assessed with an electrochemiluminescence immunoassay (ECLIA) on fully-automated COBAS 6000 e601 module analyzer (Roche Diagnostics GmbH, Penzberg, Germany), according to the manufacturer’s specifications and using proprietary reagents. After blood sampling, a prostatic massage was performed, always from the same urologist and consisting in three digital pressure per lobe, so 20–30 ml of urine were then collected in a sterile urine container (Nalgene, Rochester, NY, USA) and transferred into a specific transport tube (Progensa PCA3 Urine Specimen Transport Kit, San Diego, CA, USA) to be stored at −80°C until processing. The PROGENSA PCA3 assay (Gen-Probe Inc., San Diego, CA, USA) was used to evaluate the PCA3 and PSA mRNA expression levels in urine samples, in order to calculate the PCA3 score as the ratio of PCA3 to PSA mRNA × 1000. Both urine and serum samples were collected and processed at the Clinical Pathology Laboratories of the Regina Elena National Cancer Institute. After samples testing, all patients gradually performed a saturation prostatic biopsy. All tissue samples were collected and evaluated from the Pathological Anatomy Unit of the Regina Elena National Cancer Institute. If more than one neoplastic focus was detected in the same tumor, the highest Gs was reported.

### Statistical analyses

The association between variables was tested by Pearson's Chi-square test or Fisher’s Exact test, when appropriate. The continuous data as mean and standard deviation or median and range was reported. Binary data was reported as frequency and percentage values. Kruskal-Wallis or Mann–Whitney (adjusted for multiple comparison, when appropriate) were used for the comparisons. A p-value ≤ 0.05 was considered statistically significant.

The receiver operating characteristic (ROC) curve analysis was performed in order to find possible optimal cut-offs capable of splitting patients in two groups and for assess models predictive accuracy through the estimation of the area under the curve (AUC), providing specificity, sensitivity, negative and positive predictive value (NPV and PPV), and the 95% confidence interval (CI) for all possible threshold values and differences between curves. The SPSS®(21.0) statistical program was used for all the analyses.

## Results

**Figure 1:**
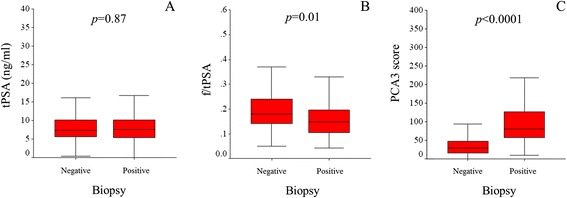
**tPSA (A), f/tPSA (B) and PCA3 score (C) values for patients negative and positive for PCa.**

Out of the 407 men enrolled, all were tested for tPSA, fPSA, and PCA3; moreover, all of them performed a subsequent biopsy that revealed 195 (48%) tumors. For both the PCa and non-PCa groups, data concerning the median age, tPSA, f/tPSA and PCA3 values were summarized in Table 1. Comparing PCa *versus* non-PCa men, no difference in tPSA values were found (*p* = 0.87), while men with PCa showed a lower median f/tPSA (*p* = 0.01) and a significantly higher median of the PCA3 score (*p* < 0.0001), compared to men without PCa (Figure 1). No association with age was found.

**Table 1:** **Number of PCa-positive and PCa-negative patients and evaluation of the related distribution in terms of median age, tPSA, f/tPSA and PCA3 score values**

	**PCa**	**non-PCa**	***p*** **value**
**Number (%)**	195 (48)	212 (52)	/
**Age**	71 ± 27	69 ± 31	0.33
**(median ± SD)**			
**tPSA (ng/ml)**	7.53 ± 4.88	7.34 ± 5.87	0.87
**(median ± SD)**			
**f/tPSA**	0.15 ± 0.07	0.18 ± 0.07	0.01
**(median ± SD)**			
**PCA3 score**	82 ± 45	33 ± 26	<0.0001
**(median ± SD)**

**Figure 2:**
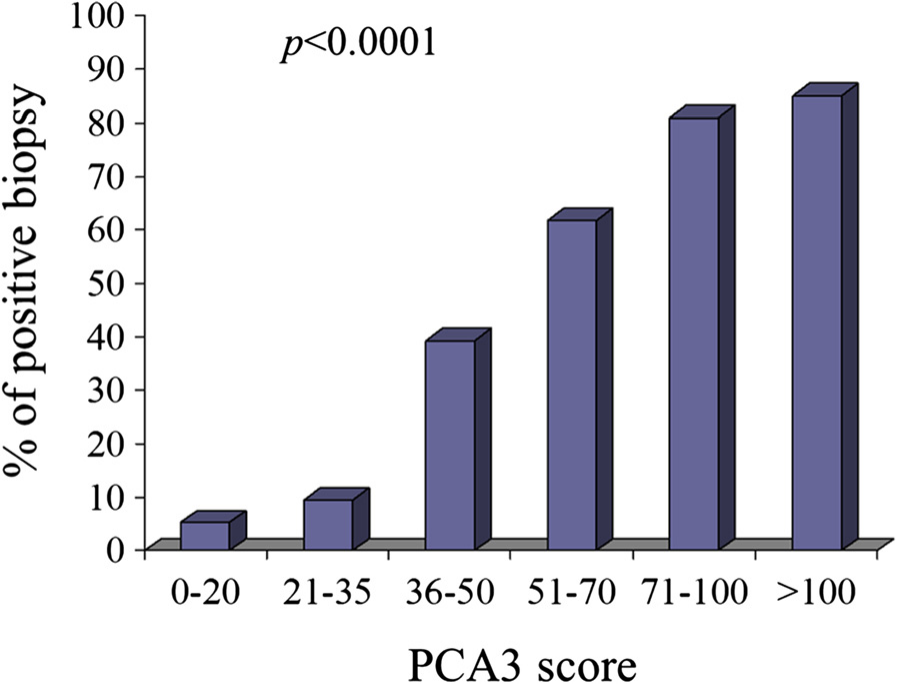
**Relationship between PCA3 score and the percentage of positive biopsies.**

To further evaluate the clinical significance of the PCA3 test, six intervals of PCA3 score values *versus* biopsy outcomes were chosen (Figure 2). Specifically, PCA3 score values were parted in increasing ranges (0–20, 21–35, 36–50, 51–70, 71–100 and >100) so the number of PCa-positive biopsies for each interval was evaluated. The probability to find a positive biopsy strongly correlates with the PCA3 test, as the probability to find a PCa-positive biopsy is higher at increased PCA3 score values (*p* < 0.0001).

In order to characterize the best cutoff of the PCA3 test, the number of true negative (TN), true positive (TP), false negative (FN), and false positive (FP) at different PCA3 scores were evaluated. Consequently, sensitivity and specificity, for each considered threshold, as well as the PPV and NPV were calculated. Considering our cohort, 35 overcomes 20 as PCA3 score cutoff, because a better balance between sensitivity and specificity, as well as higher PPV and NPV, were observed. However, the best result was obtained from a PCA3 score threshold of 51, that showed the best sensitivity, specificity, PPV and NPV values (Table 2).

**Table 2:** **Sensitivity, specificity, positive predictive value (PPV) and negative predictive value (NPV) of different PCA3 score cutoff**

	**PCA3 score cutoff**		
	**20**	**35**	**51**
**Sensitivity**	97.9	94.9	82.1
**Specificity**	33.3	60.1	79.3
**PPV**	47.8	68.5	78.4
**NPV**	57.4	92.8	82.8

**Figure 3:**
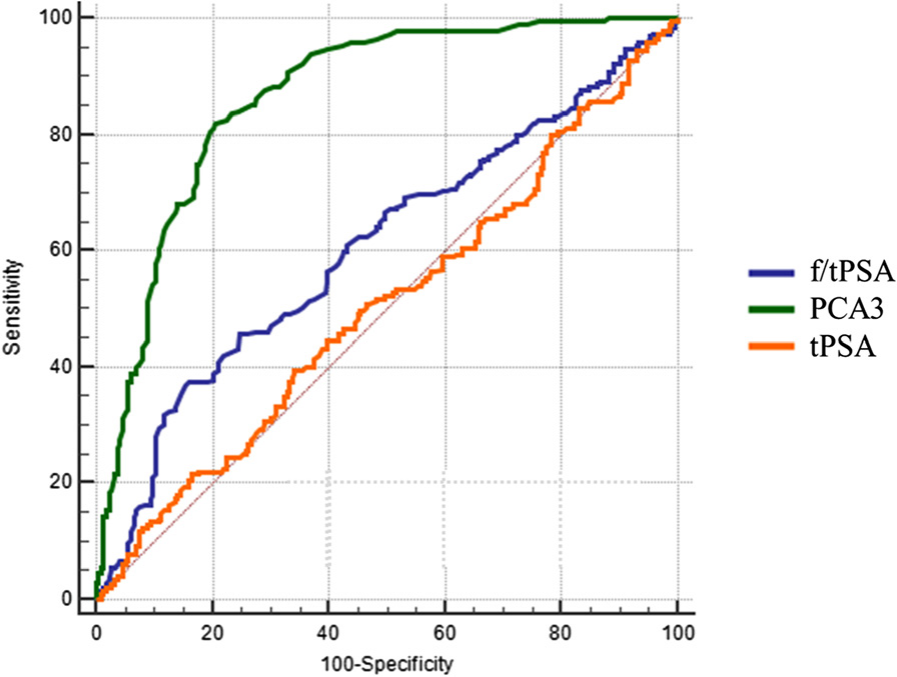
**ROC analysis with evaluation of the corresponding AUC for tPSA (0.505), f/tPSA (0.607) and PCA3 score (0.865).**

In addition, in order to compare the diagnostic performance of the PCA3 and PSA tests, a ROC analysis was performed (Figure 3). The area AUC was found to be higher for the PCA3 test (0.865) when compared to both tPSA (0.505) and f/tPSA (0.607).

Finally, the association between the PCA3 score and the tumor aggressiveness, expressed in terms of Gs score, was investigated (Table 3). The evaluation of the histologic grade was perfectly assessable on 114 PCa men. The tumor aggressiveness was split in two classes: Gs ≤ 6 (that includes the lower grades) and Gs ≥ 7 (representing the most clinically significant cases). The PCA3 score threshold of 51 (optimal for our cohort), was exceeded from the 69% of men with Gs ≤ 6, but this percentage was significantly higher (87.5%) for men with Gs ≥ 7 (*p* = 0.02).

**Table 3:** **Correlation between tumor aggressiveness, expressed in terms of Gleason score (Gs), and the PCA3 score (** ***p*** **= 0.02) in a subgroup of patients with PCa assessable histological characterization (** ***n*** **= 114)**

	**PCA3 score**	
	**≤51**	**>51**
**Gs ≤ 6 (%)**	13 (31)	29 (69)
**Gs ≥ 7 (%)**	9 (12.5)	63 (87.5)

## Discussion

The PSA limitations in PCa detection and classification are well established [13,14]. Hereupon, the risk to underestimate patients with PCa because of normal PSA levels, and, more often, to guide patients toward specialized medical practices attempting to detect a small percentage of clinically significant cancers, is very high. Moreover, it has been shown how PSA fails to predict the lethal forms of PCa [15]. Therefore, many independent studies aimed to find and to validate new PCa biomarkers are being performed.

The present study is based on an Italian cohort of 407 men with one or more previous negative biopsies; all of them, belonging to a high risk population for PCa, were addressed to a saturation prostatic biopsy after the PCA3 test. This study succeeded in demonstrating that the PCA3 test is a more sensitive test than the tPSA and the f/tPSA tests in discriminating patients with and without PCa (Table 1 and Figure 1). In fact, for our cohort, the median tPSA value was similar between the two subgroups (*p* = 0.87), while a significant difference was found for the f/tPSA (*p* = 0.01); however, the best result was obtained considering the different distribution of the PCA3 score (*p* < 0.0001) between PCa and non-PCa patients.

Although the PCA3 test seems to improve the probability to detect PCa, it is still unclear whether a not-optimal DRE can give false negative values of the PCA3 score, as well as if this test is able to detect a neoplasia at its very initial stage; on the other hand, some reports suggest that PCA3-mRNA can be also detected in HGPIN lesions [16-18]. Although in this study LGPIN and HGPIN reports were classified as negative, the present data support the hypothesis that the probability to find a PCa gets higher when the PCA3 score increases. At a low PCA3 score, in fact, the percentage of subjects with PCa was small (5.3% for PCA3 score between 0 and 20), while the percentage increased steadily to reach the maximum when the PCA3 score exceeded 100 (*p* < 0.0001); in this case, in fact, PCa was found in 79% of patients (Figure 2).

One of the major opened questions about the PCA3 test, on the other side, regards the optimal cutoff useful to discriminate patients with and without PCa. The optimal threshold proposed by Gen-Probe Inc., using the PROGENSA PCA3 assay, was 35, but several studies suggested that this value could be modified, getting lower or even higher, in a way that is probably dependent on the population features. In this respect, the cutoff value of 20 seems to increase the PCA3 test sensitivity without affecting the specificity [19-24]. Some studies demonstrated that PCA3 is effective only after the first negative biopsy, however, a recently published meta-analysis showed that PCA3 can be used for repeat biopsy to improve accuracy of PCa detection, since a large number of unnecessary biopsies can be avoided by using a PCA3 score cutoff of 20 [12,25]. To assess the best PCA3 score value, useful to discriminate those at a tumor stage, the most commonly used thresholds were examined. In our cohort, in which a division between men with one or more previous negative biopsies was not prevented, the lowest specificity was found for 20 (33.3%) when compared to 35 (60.1%), while the sensitivity resulted very similar (97.9% and 94.9%, respectively). Even if a threshold of 35 showed a better balance between sensitivity and specificity, the best performance was reached considering a threshold of 51, showing sensitivity and specificity of 82.1% and 73.3%, respectively (Table 2). An optimal cutoff higher than 35 was found also in other independent prospective studies, where it showed the ability to prevent a larger number of unnecessary biopsies, highlighting more firmly on those patients who need a fast treatment [22,23,26]. These results were confirmed by the ROC analysis, as comparing the area under the curve for PCA3, tPSA, and f/tPSA tests we found values of 0.865, 0.505 and 0.607, respectively. These data indicate that the PCA3 test showed the best performance for the PCa diagnosis for our cohort of men (Figure 3).

Lastly, a possible correlation between the PCA3 score and the tumor aggressiveness, expressed in terms of Gs, was investigated. Subjects with organ-confined PCa and Gs ≥ 7 have a worst prognosis than those with Gs ≤ 6, even following radical prostatectomy or radiation therapy [27-29]. To recognize a low grade from a more aggressive PCa is therefore essential for therapeutic purposes, but currently the only way to discriminate patients with low or high grade PCa is to perform a biopsy. The possibility of using the PCA3 test as a prognostic marker is desirable, but the possibility to evaluate tumor aggressiveness by the PCA3 test is openly debated [17,21,23,26,30-34]. Indeed, the wide range of results obtained in previous studies may be due to different experimental conditions and may reflect the selected cohort features. In fact, the use of urine sediments or whole urine samples, collected before or without a previous DRE, can give rise to different results that are not often comparable in judging the prognostic value capabilities of the PCA3 test. On the other hand, the characteristics of the screened population could be important, indeed the choice to enroll only patients with a certain risk for PCa, or depending on the number of previous biopsies, can drive data towards an easier or less easy association between the result of the PCA3 test and the tumor aggressiveness.

The patients enrolled in this study were selected according to the presence of persistent risk factors for PCa with at least a previous negative biopsy. We evaluated, among patients with an assessable tumor grading (*n* = 114), those who exceeded the PCA3 score value of 51 (optimal for our cohort) showing, at the same time, a low grade PCa, *i.e.* Gs ≤ 6, or a higher grade PCa, represented by Gs ≥ 7 (Figure 3). For our cohort of men, a correlation between the PCA3 level and the PCa grading was actually found; indeed, the percentage of patients with a PCA3 score higher than 51 and a Gs ≤ 6 was 69%, while the percentage of patients with a PCA3 score higher than 51 and a Gs ≥ 7 (87.5%) was significantly higher (*p* = 0.02). These data strengthen the hypothesis that the PCA3 test could recognize, among PCa subtypes, those more aggressive that may benefit from the resolutive radical prostatectomy surgery.

## Conclusions

The present study was conducted on subjects with at least a previous negative prostatic biopsy and with two or more persistent risk factors for PCa, resulting therefore good candidates for a further biopsy. Here, we report that the PCA3 score shows a great diagnostic accuracy compared to both tPSA and f/tPSA tests; moreover, a high PCA3 score corresponds to an increased probability to find a positive biopsy. Our data suggest that the PCA3 test could predict a PCa and allow urologists to more easily select, among high-risk patients, those who may benefit from a saturation prostatic biopsy. Even more interesting is the finding of a correlation between PCA3 score and tumor aggressiveness, expressed in terms of Gleason score, that strengthened the hypothesis of PCA3 as an effective prognostic marker, able to discriminate, among cancers, those less significant that may directly enter the active surveillance protocols, lowering the economic effort for PCa diagnosis supported from public health.

## Abbreviations

ASAP: Atypical acinar proliferation
AUC: Area under the curve
BPH: Benign prostatic hyperplasia
DRE: Digital-rectal examination
ECLIA: Electrochemiluminescence immunoassay
f/tPSA: fPSA/tPSA ratio
FN: False negative
FP: False positive
fPSA: Free PSA
Gs: Gleason score
HGPIN: High grade prostatic intraepithelial neoplasia
LGPIN: Low-grade prostatic intraepithelial neoplasia
NPV: Negative predictive value
PCa: Prostate cancer
PCA3: Prostate cancer gene 3
PPV: Positive predictive value
PSA: Prostate specific antigen
ROC: Receiver operating characteristics
TMA: Transcription mediated amplification
TN: True negative
TP: True positive
tPSA: Total PSA

## References

[1] Siegel R, Naishadham D, Jemal A (2013). Cancer statistics, 2013. CA Cancer J Clin.

[2] Barba M, Yang L, Schünemann HJ, Sperati F, Grioni S, Stranges S (2009). Urinary estrogen metabolites and prostate cancer: a case–control study and meta-analysis. J Exp Clin Cancer Res.

[3] Ribeiro R, Monteiro C, Cunha V, Oliveira MJ, Freitas M, Fraga A (2012). Human periprostatic adipose tissue promotes prostate cancer aggressiveness in vitro. J Exp Clin Cancer Res.

[4] Sofra M, Antenucci A, Gallucci M, Mandoj C, Papalia R, Claroni C (2014). Perioperative changes in pro and anticoagulant factors in prostate cancer patients undergoing laparoscopic and robotic radical prostatectomy with different anaesthetic techniques. J Exp Clin Cancer Res.

[5] Croswell JM, Kramer BS, Crawford ED (2011). Screening for prostate cancer with PSA testing: current status and future directions. Oncology (Williston Park).

[6] Croswell JM, Kramer BS, Crawford ED (2011). Screening for prostate cancer with PSA testing: current status and future directions. Oncology.

[7] Matlaga BR, Eskew LA, McCullough DL (2003). Prostate biopsy: indications and technique. J Urol.

[8] Raja J, Ramachandran N, Munneke G, Patel (2006). Current status of transrectal ultrasound-guided prostate biopsy in the diagnosis of prostate cancer. Clin Radiol.

[9] Nogueira L, Corradi R, Eastham JA (2010). Other biomarkers for detecting prostate cancer. BJU Int.

[10] Bussemakers MJ, van Bokhoven A, Verhaegh GW, Smit FP, Karthaus HF, Schalken JA (1999). DD3: a new prostate-specific gene, highly overexpressed in prostate cancer. Cancer Res.

[11] Day JR, Jost M, Reynolds MA, Groskopf J, Rittenhouse H (2011). PCA3: from basic molecular science to the clinical lab. Cancer Lett.

[12] Luo Y, Gou X, Huang P, Mou C (2014). The PCA3 test for guiding repeat biopsy of prostate cancer and its cut-off score: a systematic review and meta-analysis. Asian J Androl.

[13] Andriole GL, Crawford ED, Grubb RL, Buys SS, Chia D, Church TR (2009). PLCO Project Team. Mortality results from a randomized prostate-cancer screening trial. N Engl J Med.

[14] Schröder FH, Hugosson J, Roobol MJ, Tammela TL, Ciatto S, Nelen V (2009). Screening and prostate-cancer mortality in a randomized European study. N Engl J Med.

[15] Fall K, Garmo H, Andrèn O, Bill-Axelson A, Adolfsson J, Adami HO (2007). Scandinavian Prostate Cancer Group Study No. 4. Prostate-specific antigen levels as a predictor of lethal prostate cancer. J Natl Cancer Inst.

[16] Morote J, Rigau M, Garcia M, Mir C, Ballesteros C, Planas J (2010). Behavior of the PCA3 gene in the urine of men with high grade prostatic intraepithelial neoplasia. World J Urol.

[17] Auprich M, Bjartell A, Chun FK, de la Taille A, Freedland SJ, Haese A (2011). Contemporary role of prostate cancer antigen 3 in the management of prostate cancer. Eur Urol.

[18] Montironi R, Mazzucchelli R, Lopez-Beltran A, Scarpelli M, Cheng L (2011). Prostatic intraepithelial neoplasia: its morphological and molecular diagnosis and clinical significance. BJU Int.

[19] Hessels D, Klein Gunnewiek JMT, van Oort I, Karthaus HFM, van Leenders GJL, van Balken B (2003). DD3(PCA3)-based molecular urine analysis for the diagnosis of prostate cancer. Eur Urol.

[20] Van Gils MP, Hessels D, van Hooij O, Jannink SA, Peelen WP, Hanssen SL (2008). The time-resolved fluorescence-based PCA3 test on urinary sediments after digital rectal examination; a Dutch multicenter validation of the diagnostic performance. Clin Cancer Res.

[21] Haese A, de la Taille A, van Poppel H, Marberger M, Stenzl A, Mulders PF (2008). Clinical utility of the PCA3 urine assay in European men scheduled for repeat biopsy. Eur Urol.

[22] Bollito E, De Luca S, Cicilano M, Passera R, Grande S, Maccagnano C (2012). Prostate cancer gene 3 urine assay cutoff in diagnosis of prostate cancer: a validation study on an Italian patient population undergoing first and repeat biopsy. Anal Quant Cytol Histol.

[23] Filella X, Foj L, Milà M, Augé JM, Molina R, Jiménez W (2013). PCA3 in the detection and management of early prostate cancer. Tumour Biol.

[24] Gittelman MC, Hertzman B, Bailen J, Williams T, Koziol I, Henderson RJ (2013). PCA3 molecular urine test as a predictor of repeat prostate biopsy outcome in men with previous negative biopsies: a prospective multicenter clinical study. J Urol.

[25] Goode RR, Marshall SJ, Duff M, Chevli E, Chevli KK (2013). Use of PCA3 in detecting prostate cancer in initial and repeat prostate biopsy patients. Prostate.

[26] van Poppel H, Haese A, Graefen M, de la Taille A, Irani J, de Reijke T (2012). The relationship between Prostate CAncer gene 3 (PCA3) and prostate cancer significance. BJU Int.

[27] Heidenreich A, Bellmunt J, Bolla M, Joniau S, Mason M, Matveev V (2011). EAU guidelines on prostate cancer. Part 1: screening, diagnosis, and treatment of clinically localised disease. Eur Urol.

[28] Albertsen PC, Moore DF, Shih W, Lin Y, Li H, Lu-Yao GL (2011). Impact of comorbidity on survival among men with localized prostate cancer. J Clin Oncol.

[29] van den Bergh RC, Giannarini G (2014). Prostate cancer: surgery versus observation for localized prostate cancer. Nat Rev Urol.

[30] Hessels D, van Gils MP, van Hooij O, Jannink SA, Witjes JA, Verhaegh GW (2010). Predictive value of PCA3 in urinary sediments in determining clinico-pathological characteristics of prostate cancer. Prostate.

[31] Durand X, Xylinas E, Radulescu C, Haus-Cheymol R, Moutereau S, Ploussard G (2012). The value of urinary prostate cancer gene 3 (PCA3) scores in predicting pathological features at radical prostatectomy. BJU Int.

[32] Liss MA, Santos R, Osann K, Lau A, Ahlering TE, Ornstein DK (2011). PCA3 molecular urine assay for prostate cancer: association with pathologic features and impact of collection protocols. World J Urol.

[33] Auprich M, Chun FK, Ward JF, Pummer K, Babaian R, Augustin H (2011). Critical assessment of preoperative urinary prostate cancer antigen 3 on the accuracy of prostate cancer staging. Eur Urol.

[34] Nakanishi H, Groskopf J, Fritsche HA, Bhadkamkar V, Blase A, Kumar SV (2008). PCA3 molecular urine assay correlates with prostate cancer tumor volume: implication in selecting candidates for active surveillance. J Urol.

